# Human Parechovirus Genotypes -10, -13 and -15 in Pakistani Children with Acute Dehydrating Gastroenteritis

**DOI:** 10.1371/journal.pone.0078377

**Published:** 2013-11-12

**Authors:** Muhammad Masroor Alam, Adnan Khurshid, Shahzad Shaukat, Muhammad Suleman Rana, Salmaan Sharif, Mehar Angez, Nadia Nisar, Muhammad Naeem, Syed Sohail Zahoor Zaidi

**Affiliations:** 1 Department of Biotechnology, Quaid-i-Azam University, Islamabad, Pakistan; 2 Department of Virology, National Institute of Health, Islamabad, Pakistan; University of Kansas Medical Center, United States of America

## Abstract

Human parechoviruses are known to cause asymptomatic to severe clinical illness predominantly respiratory and gastroenetric infections. Despite their global prevalence, epidemiological studies have not been performed in Pakistan. In this study, we retrospectively analyzed 110 fecal specimen and found 26 (24%) positive for viral RNA with HPeV-10 (n = 3, 23%), HPeV-13 (n = 4, 31%) and HPeV-15 (n = 6, 46%) genotypes. Clinical features of patients with different HPeV genotypes were compared. All HPeV positive children were aged ≤4 years (mean 13.92 months). The male-to-female ratio was 1: 1.17 (46.2 vs 53.8%) with significant association (*p = .031*) to HPeV infectivity. HPeV-10 and -13 were found during summer while HPeV-15 was only detected during late winter season. Disease symptoms were more severe in children infected with HPeV-10 and -13 as compared to HPeV-15. Fever and vomiting were observed in 100% cases of HPeV-10 and -13 while only 17% patients of HPeV-15 had these complaints. Phylogenetic analyses showed that HPeV-10, -13 and -15 strains found in this study have 9–13%, 16.8% and 21.8% nucleotide divergence respectively from the prototype strains and were clustered to distinct genetic lineages. This is the first report of HPeV-15 infection in humans although first identified in rhesus macaques. The arginine-glycine-aspartic acid (RGD) motif present at the C-terminal of VP1 responsible for the viral attachment to cellular integrins was not found in all of these strains. In conclusion, these findings enhance our knowledge related to the epidemiology and genetic diversity of the HPeV in Pakistan and support the need for continued laboratory based surveillance programs especially in infants and neonatal clinical settings. Further, the parechovirus pathogenesis, cross-species transmission and disease reservoirs must be ascertained to adopt better prevention measures.

## Introduction

Human Parechovirus belongs to family *picornaviridae* within the genus *parechovirus* and contains a single stranded RNA genome encapsidated in a non-enveloped icosahedral capsid protein coat. The prototypes of this virus, HPeV-1 and HPeV-2, were initially known as echo-22 and echo-23, under the genus enterovirus but were re-classified into a separate genus on the basis of their genome organization and biological properties [1], [2]. Since their discovery in 1956 [3], several new genotypes have been identified within the past few years. To date, there have been 16 reported HPeV genotypes (www.picornaviridae.com/parechovirus/hpev/hpev.htm) although published data is available for only 12 genotypes 1–8, 10, 11, 14 and 12 [4].

HPeVs have been recognized as an important pediatric viral pathogen causing mild to severe infections including gastroenteritis, respiratory infections, encephalitis and flaccid paralysis in children under 5 years of age [5], [6]. HPeV-1 is often isolated from children with diarrhea and gastroenteritis [7]. HPeV-3 has been found associated with transient paralysis, diarrhea [7] and neonatal sepsis [8]. HPeV-4 was isolated from a 5 year old patient with lymphadenitis [9]. HPeV-6 was identified in a 1 year old girl with Reye syndrome [10]. HPeV-7 was isolated from a patient with non-polio acute flaccid paralysis in Pakistan [11]. HPeV-8 was identified in a patient with enteritis in Salvador, Brazil [11]. HPeV-10 and -11 were identified in patients with acute gastroenteritis in Sri Lanka [12], [13]. HPeV-12 was detected in 20 months old child with diarrhea and paralysis [4] while HPeV-14 was detected from a febrile child in the Netherlands [14].

The studies about the spectrum of causative agents causing acute gastroenteritis have never been conducted in Pakistan except few focused only on rotavirus in the recent years [15]–[17]. Human Parechovirus genotypes -1, -5, -6 and -7 have been previously identified from acute flaccid paralysis cases in Pakistan through metagenomic approach while HPeV-12 was recently identified in a Pakistani child with diarrhea and paralysis [4], [11]. To the best of our knowledge, comprehensive epidemiological studies focused on the prevalence of human parechovirus in hospitalized children presented with acute gastroenteritis have never been conducted in Pakistan. We here report the epidemiological and virological information obtained from retrospective analysis of stool samples collected during 2008. Due to their possible role in gastroenteritis, the molecular properties and genetic diversity of parechovirus strains were studied in relation to their clinical significance.

## Materials and Methods

In this study, stool samples from 110 children below five years were collected during January to December 2008. The children were hospitalized if fulfilled the case definition criteria of gastroenteritis. The case definition of gastroenteritis was ≥3 loose stools in 24 hours, vomiting ≥3 times in 24 hours, loose stools with two additional symptoms or vomiting with two additional symptoms. Additional symptoms included diarrhea, vomiting, nausea, fever, abdominal pain, abdominal cramps, and blood or mucus in stool. The study concept and design was approved through the Pakistan's National Institute of Health Internal Review Board. All samples were collected after informed and written consent from the patient's parents/guardians at the time of sample collected on a standard questionnaire approved by the NIH Internal review board.

The collected samples were analyzed for the presence of HPeV RNA directly by real time PCR as described earlier [18]. Although this 5′UTR based real-time assay detects both HPeV and Ljungan virus RNA, HPeV was confirmed by amplification of 760 bp fragment within VP1 gene positioned from nt. 2332 to 3090 according to the prototype strain Harris (GenBank accession number L02971) using the primers VP1-parEchoF1 (5′-CCAAAATTCRTGGGGTTC-3′) and VP1-parEchoR1 (5′-AAACCYCTRTCTAAATAWGC-3′) as described by Benschop et al [19]. The PCR amplicons of VP1 gene were purified and sequenced in both directions using a BigDye Terminator cycle sequencing kit v3.1 (Perkin Elmer-Applied Biosystems, Inc., Foster City, CA). The sequence data was collected by an ABI Prism genetic analyzer 3100, edited using Sequencher v.4.1 (Gene Codes Inc., Ann Arbor, MI, USA) and the phylogenetic tree was reconstructed using MEGA 4.0.

HPeV genotypes were determined on the basis of sequence analysis of VP1 gene fragment with threshold of ≥77% and ≥87% nucleotide and amino acid identity respectively [20]. Phylogenetic analyses were performed in comparison to the strains belonging to different genotypes and geographical regions as retrieved from GenBank. Evolutionary tree and distances (number of base substitutions per site) were generated by Neighbor Joining method with Kimura-2 parameter using MEGA 4.0 (http://megasoftware.net/). The percentage of replicate trees in which the associated taxa clustered together in the bootstrap test (1000 replicates) is shown next to the branches. The GenBank accession numbers, country, year of sample collection and respective genotype information has been given where available. The HPev strains found in this study have been submitted to GenBank under the accession Nos. KF626450-62.

The descriptive statistical analysis like frequencies, percentages, ratio, mean, median and range were carried out for age, gender, duration of symptoms and treatment. The analysis of variance (ANOVA) was performed for the comparison of various demographic features among HPeV-10, HPeV-13 and HPeV-15 infected patients. The p-value less than 0.05 (two sided) were considered statistically significant. The association between different HPeV genotypes and disease severity was established through Fisher's Chi-square test (χ2). The statistical analyses were performed by using SPSS ver. 18.

## Results

From January-December 2008, a total of 26/110 (24%) clinical samples were determined to be positive for parechovirus by real-time RT-PCR in our laboratory. Of those, HPeV genotypes were identified in 13/26 (50%) samples. The majority of the isolates (n = 6) could be identified as HPeV-15 (46%). In addition, (n = 4) isolates were genotyped as HPeV-13 (31%) and (n = 3) isolates were identified as HPeV-10 (23%). Hundred percent of all HPeV cases reported during this time period were survived after Oral Rehydration therapy (ORT) and/or Intravenous rehydration therapy.

### Demographical Features of HPeV positive Cases

**Figure 1:**
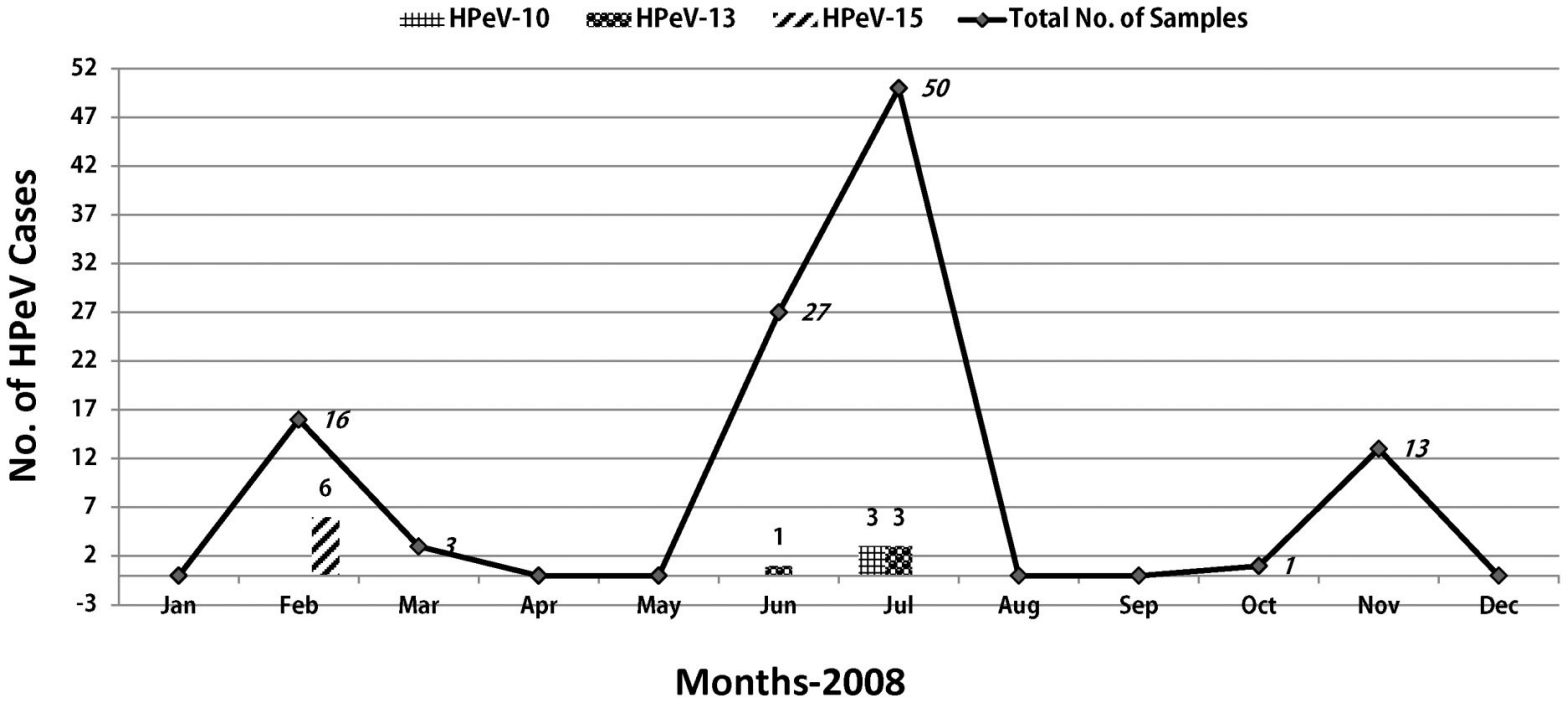
The distribution of HPeV infections in Pakistan during 2008 is presented with months given on X-axis. The data labels on Y-axis indicate the number of total and positive samples for each genotype across the year. Total number of cases is indicated by solid black line indicating typical peak season for gastroenteritis in summer (monsoon/rainy season) months. The number of cases positive for each genotype is indicated with different bar-style.

All of the 110 children presented with gastroenteritis were aged ≤4 years (mean 13.92 months; median 8 months; range 1 month to 4 years), whereas the mean age for HPeV positive cases was 13.9±13.2 months (median 8 months; range 1 to 40 months) (Table 1). HPeV-10 and -13 infections were more frequently found in females (50% and 100% respectively) as compared to males; however, 83% infections with HPeV-15 genotypes were found in males. All the cases with HPeV-15 were identified in winter while infections with HPeV-10 and -13 were reported during the monsoon months of June and July (Figure 1).

**Table 1:** Comparative analysis of clinical variables (quantitative) among children positive for human parechovirus (HPeV) infection in Pakistan using ANOVA.

Characteristics	Mean±S.D	Range	*p-value*
Age (months)	13.9±13.2	(1–40)	*0.345*
	8		
Duration of Symptoms (days)	2.5±1.6	(1–5)	*0.016* *
	3		
Vomiting Episodes per 24 hours	3.5±3.2	(0–8)	*0.003* *
	6		
Vomiting Duration (days)	1.8±2	(0–6)	*0.008* *
	2		
Diarrhea episodes per 24 hours	7.8±1	(6–10)	*0.019* *
	8		
Diarrhea Duration (days)	2.9±2.3	(1–9)	*0.048* *
	3		
Duration of Treatment (days)	1.3±1.3	(0–3)	*0.001* *
	2		

Values with Mean±SD, Median and Range are given for positive cases. The data values with asterisk.

* indicates significant *p*-values.

The mean duration of HPeV symptoms was recorded as 2.5±1.6 days. Among the HPeV positive cases, the number of vomiting and diarrhea episodes per 24 hours was found to be 3.5±3.2 and 7.8±1 respectively. The symptoms of diarrhea persisted for 3±2.3 days whereas vomiting continued for 1.8±2 days. Upon hospitalization, all of the HPeV infected children were recovered within 1–3 days of oral and/or intravenous rehydration therapy.

### Comparison of HPeV Genotypes and Diseases Severity

Infections with different HPeV genotypes were compared for disease severity and clinical symptoms using Fisher's Chi-square test (χ^2^) (Table 2). Among the three genotypes, fever, vomiting, diarrhea and ORT treatment were found statistically significant (*p<0.05*). Disease symptoms were more severe in children infected with HPeV-10 and -13 as compared to HPeV-15. For instance, fever and vomiting were observed in 100% cases of HPeV-10 and -13 while only 17% patients of HPeV-15 had these complaints. In contrast, all HPeV infected children, irrespective of the genotype, were presented to the hospital with the complaints of diarrhea although the duration was prolonged among HPeV-13 cases (4.75±3 days). Similarly, the duration of vomiting remained for 3±1.48 days in HPeV-10 and -13 cases as compared to HPeV-15 cases (0.17±0.40). To conclude, disease symptoms were significantly more severe in HPeV-10 and -13 cases as compared to HPeV-15 cases (*p<0.05*).

**Table 2:** Comparison of demographic and clinical details of patients infected with different genotypes of human parechovirus.

Characteristics	Mean±S.D	Range	*p-value*
Age (months)	13.9±13.2	(1–40)	*0.345*
	8		
Duration of Symptoms (days)	2.5±1.6	(1–5)	*0.016* *
	3		
Vomiting Episodes per 24 hours	3.5±3.2	(0–8)	*0.003* *
	6		
Vomiting Duration (days)	1.8±2	(0–6)	*0.008* *
	2		
Diarrhea episodes per 24 hours	7.8±1	(6–10)	*0.019* *
	8		
Diarrhea Duration (days)	2.9±2.3	(1–9)	*0.048* *
	3		
Duration of Treatment (days)	1.3±1.3	(0–3)	*0.001* *
	2		

The data values with asterisk.

* indicates significant *p*-values. M = Male; F = Female; ORT = Oral Rehydration Therapy; IV = Intravenous.

### Molecular identification of HPeV

Thirteen (50%) out of 26 samples were successfully genotyped by VP1 analysis (Table 2). The majority of isolates were identified as HPeV-15 (46%) in addition to HPeV-13 (31%) and HPeV-10 (23%). All children with HPeV infection were survived (mean treatment days = 2). The mean age of children positive for HPeV-10, HPeV-13 and HPeV-15 was 4.33 months (range1–7 months), 14.25 months (range 6–24 months) and 18 months (range 1–40 months) respectively (*p = 0.345*; Analysis of Variance). All HPeV-13 infections were observed in boys while male to female ratio for HPeV10 and HPeV15 infections were found as 2∶1 and 1∶5 respectively (*p = 0.031*).

### Phylogenetic Analysis

**Figure 2:**
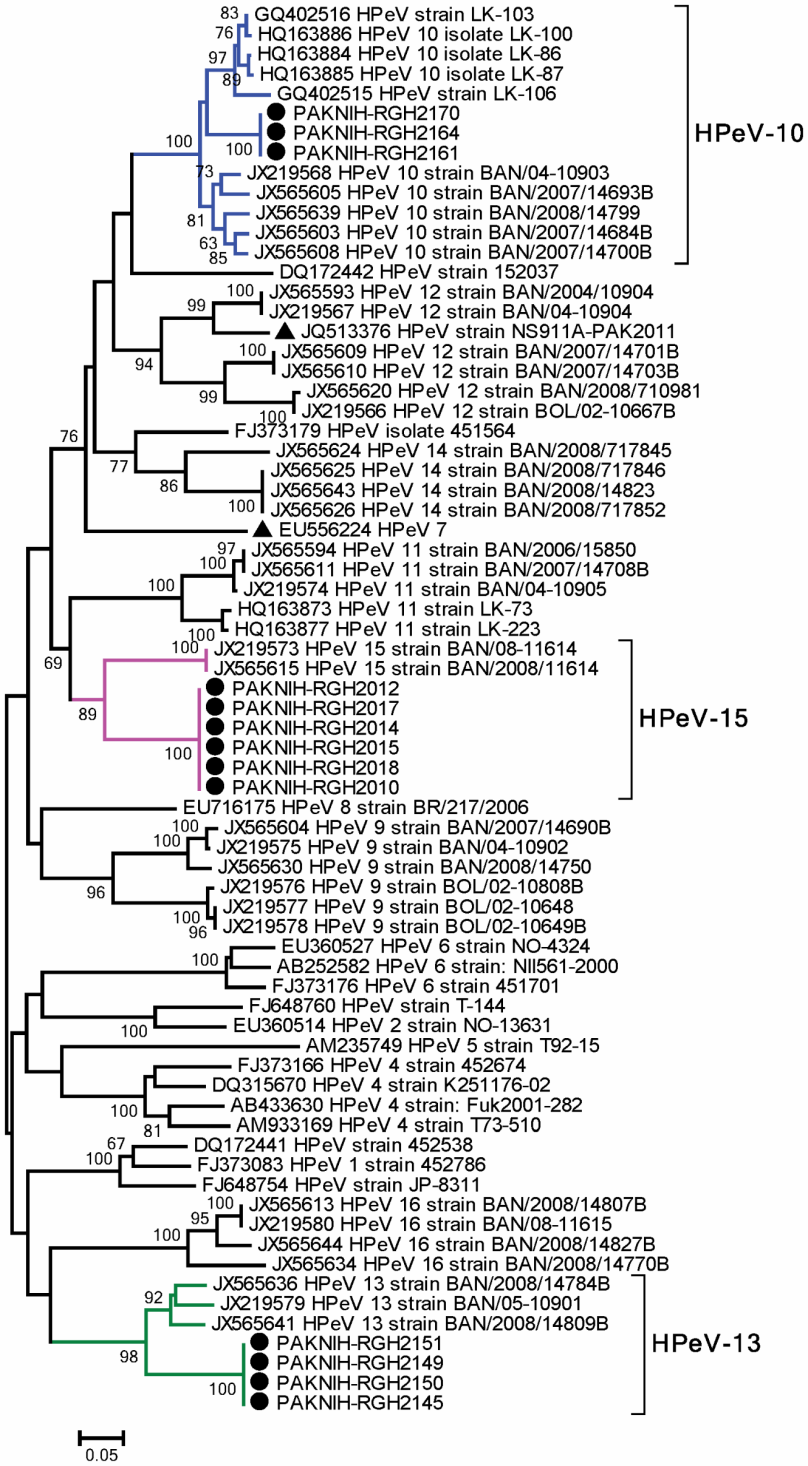
Phylogenetic analysis of human parechovirus strains identified in this study based on the partial VP1 region. The reference strains and closest match isolates detected through BLAST are given for genetic comparison. The phylogenetic tree with 1000 bootstrap replicates was reconstructed using neighbor joining method and the K-2P model through MEGA 4.0. Taxa with arrow head indicate the prototype strains within each serotype. Black circles indicate the strains found under this study in Pakistan. HPeV-12 and HPeV-7 previously reported from Pakistan are highlighted.

Nucleotide and deduced aminoacid (aa) identities were compared with the prototype sequences of respective genotypes. For HPeV-10, three strains from this study showed 100% identity to each other while 87–91% nucleotide identity was found with the prototype strains from Bangladesh (GenBank Accession No. JX219568). For HPeV-13, we detected four strains in our samples with 16.8% nucleotide and 5.1% aminoacid divergence from the prototype strain detected in Bangladesh (GenBank Accession No. JX219579). Similarly, 6 strains of HPeV-15 from Pakistan were 100% identical with 21.8% nucleotide divergence from the prototype strain reported from Bangladesh (GenBank Accession No. JX219573). Genetic analysis revealed that all HPeV viruses detected in our samples present distinct lineages than the previously known strains. The prototypes of HPeV-13 and HPeV-15 have only been detected in Bangladesh while HPeV-10 has been found in Sri Lanka as well. However, the viruses from each of these geographical regions were placed into separate lineages (Figure 2).

The RGD (Arginine-glycine-aspartic acid) motif present at the C-terminal region of VP1 gene has been previously detected in few of the HPeV types but no such motif was found in study strains of HPeV-10, -13 and -15.

## Discussion

The human parechoviruses are known to cause a variety of clinical infections including meningoencephalitis, encephalomyelitis, flaccid transient paralysis, nosocomial infection, neonatal sepsis, myocarditis, myositis, lymphadenopathy, hand-and-mouth disease, rash, fever of unknown origin, influenza-like illness, Reye's syndrome, and hemolytic-uremic syndrome [5], [7], [8], [10], [21]–[28]. With their increasing clinical significance and dynamic epidemiology, the present study was designed to retrospectively screen the fecal samples kept archived at Department of Virology, National Institute of Health, Pakistan that were collected from hospitalized children with acute gastroenteritis. These samples were found negative for rotavirus antigen using the ProSpecT™ Rotavirus Microplate Assay (Oxoid Ltd., Basingstoke Hants, UK). Out of total 110, we found 26% samples positive for HPeV using Real-time PCR targeting 5ÚTR. When compared, we found a relatively higher prevalence of HPeVs in our population than the developed countries like Germany (11.6%) and the Netherlands (13.4%) [14], [29] however, the epidemiological data from the Eastern Mediterranean countries is not available. This also indicates the improved efficiency of molecular assays for direct detection of HPeVs as compared to cell culture which is used to pre-amplify the viral titer but have disadvantages of selective growth thus reducing the viral diversities [30]. However, these findings require to re-assure the causal relationship of HPeVs in pediatric infections especially gastroenteritis through screening of matched healthy controls or antibody sero-conversion testing in the convalescent sera [31].

Majority of HPeV infections occur in children below 5 years of age with subclinical or mild infections (10, 33, 37) however, in adults above 5 years of age, only minor infection with HPeV-1 has been reported [22]. The HPeV infections in our population were found in children less than 4 years of age. The HPeV-13 infections were present in children between 6 months to 2 years. Infection with HPeV-15 was found in more diverse age group including patients between 1 month to 3.4 years of age; however HPeV-10 infections were exclusively among the children less than 1 year of age (mean age = 4 months). Infection with HPeV-10 and -15 within the first month of life indicates the lack of maternal protection and limited viral circulation in our population. This is in contrast to other HPeV genotypes where 95–99% of neonates have been found seropositive especially against the globally prevalent HPeV-1, -2 and -3 [6], [32], [33]. The extent of endemicity of new HPeV genotypes could not be established unless the serology based epidemiological surveys are conducted. The data also highlights that the clinical screenings for HPeV must be implemented especially in the pediatric units and any undiagnosed case with serious complications should be screened for HPeVs [34].

In Pakistan, majority of HPeV cases were detected during summer and post-monsoon months, however, all of the HPeV-15 infections were found in winter; a pattern similar to HPeV-1, -3, -4 and -6 which are more dominant during fall and winter in Europe and Asia [10], [30], [33]. Infection of human parechoviruses usually occur during late summer (July–August) and early winter (November–December) [35] but majority of these studies are focused on the common genotypes and the data related to the most recently identified genotypes 7 to 16 is largely unknown due to very limited number of cases reported globally. In the United Kingdom, HPeV-1 and -6 circulate throughout the year without any seasonal preference [35] whereas HPeV-3 circulation has been reported to occur as bi-annual peaks and infections with HPeV-2 have been reported sporadically [36], [37]. In Pakistan, the environmental factors may play an important role in the incidence of enteric infections during the monsoon season due to increased exposure of children to the contaminated waters. During monsoon, the rain water carrying human and animal waste pollutes the ground and surface water sources (http://www.nih.org.pk/files/Newsletter/Seasonal Awareness and Alert Letter(SAAL)24th Issue.pdf) and contribute to the outbreaks of human enteric infections [38]–[43]. Fecal-oral transmission of enteric infections including HPeV is also supported by its high viral load in the stool which may be up to 502,380 copies per ml [29]. In most of the country areas, the drinking water sources are not maintained properly, nor pre-treated, resulting to major outbreaks of enteric infections (http://en.wikipedia.org/wiki/Water_supply_and_sanitation_in_Pakistan) [14]. Therefore, a multi-focused approach including all the necessary measures ranging from proper hygiene, provision of safe drinking water and improved medical facilities must be implemented to control such infections especially in the neonates and young infants who are suffered with more severe HPeV complications and long-term sequelae than older children [44]–[46].

Since the identification of HPeV approx. 52 years ago in association with an outbreak of gastroenteritis [3], multiple studies have evaluated the role of HPeVs in gastroenteritis [35], [47]–[49]. No direct evidence of causal relationship has yet been established however HPeV types 4 and 6 have been implicated as cause of diarrhea in children [34]. Among the clinical signs, diarrhea and vomiting is found to be more commonly associated with HPeV-1 than HPeV-2 and 3 [6], [36], [50]. In Germany, HPeVs have been found to cause low-level enteric infections without any serious outbreaks [29]. In Thailand, 14.6% children (6–24 months) with acute gastroenteritis were found positive for HPeV mainly type 1, 2, 3 and 4 [50]. In Sri Lanka, 8.3% children (aged 2–26 months) hospitalized with gastroenteritis were found positive for HPeV with types 1, 3, 4, 5, 10, 11 [13]. In Japan, children aged 2 months to 15 years with acute gastroenteritis were studied and 8.1% children were infected with HPeV types 1 and 3 [51]. In a Brazilian study, HPeV types 1, 5, 6 and 8 were identified in 16.1% children with acute diarrhea [52]. HPeV-1, -3, -4, -5, -6 and -8 have been identified in Chinese children with gastroenteritis but no causal relationship was established [53]. Such findings suggest that HPeV may play a significant role in endemic cases of diarrhea among infants and younger children. Although we found HPeV in 24% of our patients enrolled with severe dehydrating gastroenteritis, the confirmed role of HPeV with such clinical complications cannot be defined and require further evidence. In 23% of our samples, HPeV-10 was identified as previously reported by Pham et al., where this type was found in two child inpatients having acute gastroenteritis at a hospital in Kandy, Sri Lanka during 2005–2006 [12] and concluded the probable role of HPeV-10 in gastroenteritis. The epidemiological and clinical data related to HPeV-13 and -15 is not available so far.

Until 2004, only two HPeV types were known, followed by identification of 14 new types within few years [54]. Despite the continued surveillance in developed countries, the new genotypes have not been found from regions other than Asia (Pakistan, Bangladesh and Sri Lanka). Multiple genotypes of parechoviruses have been reported from Pakistan; HPeV type -7 was identified in Pakistan [11], followed by HPeV-12 [4] and genotypes-10, -13, and -15 in this study. This may represent their localized geographical existence or the infections may go un-diagnosed due to lack of routine testing in clinical settings [55]. Similarly, the worldwide distribution of these types is still being defined.

The methodologies adopted for HPeV genotyping are based on sequence analysis of the hypervariable region of VP1 with threshold of ≥77% and ≥87% nucleotide and amino acid identity respectively [20]. We found 26 samples positive through real-time PCR although the typing was successful for only 13 samples. The disadvantage of typing procedures has been extensively reported and found that the VP1 assays are less sensitive than the 5ÚTR based real-time PCR [30], [50], [53], [56], [57] due to lower sensitivity or RNA degeneracy resulting in misdiagnosis of low-titer samples [58]. On the other hand, the genomic sequence variability and the frequent recombination between 5′UTR and the capsid-encoding region prevents HPeV typing through 5′UTR sequencing alone [59]. Therefore, the typing assays available to date are focused on the amplification and sequencing of VP1 gene segment. Compared to the other clinical diagnostic tools, the detection of HPeV infection is not straight forward and require specialized technical expertise and facilities. Although HPeV-1, 3, 4 and 6 have been recovered through a variety of cell lines i.e. BSC-1, CaCo2, RD-18S, LLC-MK2, Vero and HeLa [10], there is no established set of standard cell lines that can be used to isolate all HPeV genotypes especially the recently identified types 7–16. The in-vitro growth adaptation of parechoviruses is usually poor and may take up to 14–18 days in addition to the variable growth characteristics of different types [8], [60], [61] therefore, the virus isolation is not recommended for the clinical diagnosis [11], [19], [52], [59], [61]–[65].

Similarly, serotyping of viral types require cultivation of the isolate which is practically cumbersome and fails to detect new types or variants of known types [66]. The serological assays used to detect HPeV-specific antibody responses are usually time extensive and the lack of commercial reagents make them impractical for routine clinical use [67], [68]. These limitations led to the development of molecular assays that initially targeted only HPeV-1 and -2 [59], [62], [69] and/or HPeV-1 to -6 requiring multiple sets of primers [19], [29], [70]–[73] but have now been improved to target all known genotypes and have become gold standard for the detection of HPeV in clinical samples [69], [74]. In 2008, Nix et al., designed a real-time PCR with the detection threshold of 30 RNA copies [18] and found to be 100- to 1,000-fold more sensitive than virus isolation in cell culture. Although this assay was designed on the basis of available sequences for HPeV-1 to -6, but our findings substantiates its vast application in the diagnostic settings targeting all 16 genotypes known so far.

In contrast to other picornaviruses, the capsid protein, VP1, of HPeVs is found to be poorly reactive and the predominant antigenic sites of HPeV have been mapped to the N-terminal region of the VP0 protein which is not antigenic in any other genera of the *picornaviridae* family [75]. Also, the C-terminal region of VP1 protein of HPeV containing arginine-glycine-aspartic acid (RGD) motif is found to be immune-reactive. The VP1 gene of HPeV-1, -2, -4, -5 and -6 contains this motif utilized for cell-cell or cell-matrix interactions [76]–[79]. This motif is used by a number of picornaviruses including foot-and-mouth disease virus, echovirus 9 [80] and Coxsackievirus A9 for interaction with the host cell surface αvβ1 and αvβ3 integrin receptors [81]–[85]. However, no such motif has been identified for HPeV-10, -13 and -15 found in this study and provide evidence for the presence of an RGD-independent pathway of viral entry to host cells but no such pathway has yet been identified.

In our samples, the parechovirus genotype 15 has been found for the first time in human population after its only detection from rhesus macaques in Bangladesh [86]. However, many other HPeV types i.e. HPeV-1, -2, -4, -5, -12 and -14 have been reported from non-human primates (*rhesus macaque*) in Bangladesh and China [86], [87]. Similarly, in Bolivia, pigs have been found to be infected with HPeV-4 [88]. In 2013, Reuter et al., has reported two new picornaviruses; bovine hungarovirus and ovine hungarovirus, discovered in domestic animals that contain HPeV-like genetic markers in their 5′UTR [89]. In addition, a new picornavirus, Ljungan virus, has been identified from bank voles (*Clethrionomys glareolus*) in Sweden with remarkable capsid sequence homology to human parechoviruses [90]. Such a growing evidence of shared parechovirus reservoir between humans and animal species raise austere concerns of their zoonotic potential and the unidentified animal reservoir for human infections. Such findings also highlight our incomplete knowledge about the cellular tropism, virulence and cross-species transmission potential for parechoviruses [89]. Recently, in Pakistan, we have found human rotavirus G6P [1] strains that are closely resembled to bovine rotaviruses [16]. These findings highlight the risk of potential environment for the zoonotic infections in our country. Being an agricultural country, majority of Pakistani population is dependent on livestock farming which is the sole source of livelihood in many country areas. Therefore, such epidemiological studies should be extended to those country areas where animals live in close proximity to humans.

## Conclusion

Our data further expand the knowledge about the prevailing HPeV genotypes and their clinical association in children hospitalized with acute gastroenteritis. Genotypes HPeV-10, -13 and -15 are hereby reported for the first time in our population. Considerable diversity of HPeV types prevail in Pakistan, therefore, large scale epidemiological and sero-surveys should be conducted to understand their clinical relevance, epidemiology and to improve preventive measures. The infections of parechoviruses in domestic and wild animals raise several concerns about their potential zoonotic risks and need further studies.

In conclusion, HPeV infections are quite common in our population. Pakistan is among one of the Asian countries where diarrhea associated deaths are significantly higher [91]. Despite these distressing facts, viral screenings are not performed in our country hospitals as the proper laboratory facilities are not in place due to limited resources. In addition, lack of awareness among the clinicians [92] also contributes to the undiagnosed or misdiagnosed viral infections that are usually treated with unnecessary antibiotic regimen [19], [59]. This is in contrast to the developed settings like USA and the Netherlands where routine surveillance programs for HPeVs are in place for years [36]. The enormous genetic diversity and disease burden in very early age groups of our population further emphasize to prioritize the testing of HPeVs especially in the neonates and early-age infants.
