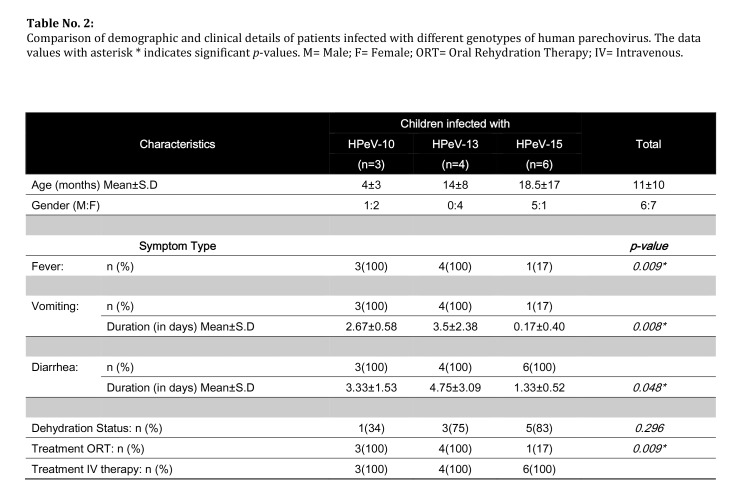# Correction: Human Parechovirus Genotypes -10, -13 and -15 in Pakistani Children with Acute Dehydrating Gastroenteritis

**DOI:** 10.1371/annotation/b77b2387-58b0-41d0-96ff-e74b9279f652

**Published:** 2013-12-20

**Authors:** Muhammad Masroor Alam, Adnan Khurshid, Shahzad Shaukat, Muhammad Suleman Rana, Salmaan Sharif, Mehar Angez, Nadia Nisar, Muhammad Naeem, Syed Sohail Zahoor Zaidi

Table 2 is missing from the published article and a duplicate of Table 1 was published in its place. The correct tables can be viewed here.

Table 1: 

**Figure:**
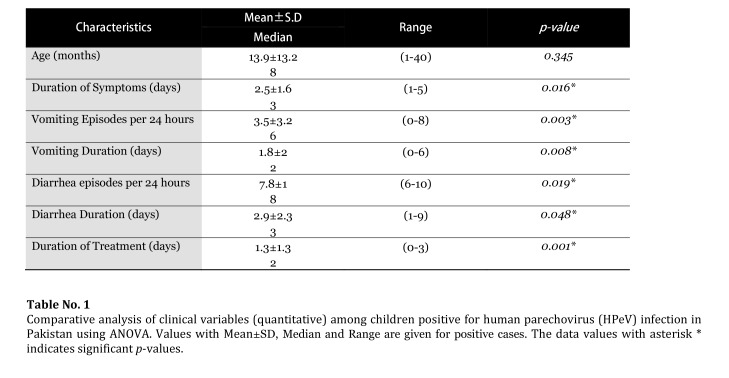


Table 2: 

**Figure:**